# Real-Time Approach to Flow Cell Imaging of *Candida albicans* Biofilm Development

**DOI:** 10.3390/jof3010013

**Published:** 2017-03-06

**Authors:** Andrew McCall, Mira Edgerton

**Affiliations:** Department of Oral Biology, University at Buffalo, Buffalo, NY 14214, USA; amccall@buffalo.edu

**Keywords:** *Candida albicans*, biofilm, flow, time-lapse

## Abstract

The ability of *Candida albicans* to form biofilms is a virulence factor that allows tissue attachment and subsequent infection of host tissues. Fungal biofilms have been particularly well studied, however the vast majority of these studies have been conducted under static conditions. Oral biofilms form in the presence of salivary flow, therefore we developed a novel flow system used for real-time imaging of fungal biofilm development. *C. albicans* wild-type (WT) cells readily attached to the substrate surface during the 2 h attachment phase, then formed heterogeneous biofilms after 18 h flow. Quantitative values for biomass, rates of attachment and detachment, and cell–cell adhesion events were obtained for *C. albicans* WT cells and for a hyperfilamentous mutant Δ*hog1*. Attachment rates of *C. albicans* WT cells were nearly 2-fold higher than *C. albicans* Δ*hog1* cells, although Δ*hog1* cells formed 4-fold higher biomass. The reduced normalized detachment rate was the primary factor responsible for the increased biomass of Δ*hog1* biofilm, showing that cell detachment rates are an important predictor for ultimate biofilm mass under flow. Unlike static biofilms, *C. albicans* cells under constant laminar flow undergo continuous detachment and seeding that may be more representative of the development of in vivo biofilms.

## 1. Introduction

*Candida albicans* is the most common source of oral and systemic fungal infections [1]. Despite improvements in treatment modalities, mortality rates for these infections remain relatively high, in part due to drug resistance [2]. Formation of biofilms by fungal cells contributes to drug resistance due to the higher cell density preventing efficient drug trafficking into cells, the presence of a protective extracellular matrix (ECM), and alterations in gene expression of cells within the biofilm [3]. Numerous in vitro studies have analyzed the growth and development of *C. albicans* biofilms on many surfaces, including acrylic, silicone, plastic, glass, and catheters [4,5,6,7]. However, these studies have largely been carried out under static conditions in which the medium overlying the biofilm lacks flow. 

*C. albicans* colonizes surfaces under dynamic flow in vivo, including the oral mucosa or denture surfaces in the presence of salivary flow. In the oral cavity, saliva is a nutrient poor, low flow, complex bodily fluid consisting of host proteins, microbiota, and various food remnants [8]. The average shear stress generated by saliva across a tooth surface has been calculated to be 0.8 dynes/cm^2^ [9]. Despite this relatively low shear stress value; it is likely that salivary flow contributes to reduced formation of *C. albicans* biofilms, as patients with xerostomia (reduced salivary flow) are at increased risk of developing oral candidiasis [10,11].

Few in vitro studies have examined the role that fluid flow may have on *C. albicans* biofilm development [12,13,14], even though colonization of *C. albicans* occurs under flow in several human niches. Flow is important for three main reasons: first, flow generates a force parallel to the surface of the substrate (shear force) that works to remove cells from the biofilm and therefore reduces the mass of the biofilm; second, flow is responsible for dispersion of detached cells and subsequent delivery of these cells to new sites; and thirdly, flow allows bulk fluid movement to deliver fresh nutrients to cells within the biofilm. This last point is particularly important for biofilm development since environmental nutrient availability is a potent signal for *C. albicans* hyphal formation [15]. Earlier studies confirm that biofilms formed under flow have different morphologies than static biofilms [16]. Under static conditions, biofilms develop through multiple temporal stages that are characterized as adherence, initiation, maturation, and dispersion. The initial founder yeast cells adhere to the substrate in the adherence phase (the first 2 h); followed by propagation of these yeast cells to form microcolonies (and in which some top-most cells form germ tubes perpendicular from the substrate) in the initiation phase (2–11 h). During the maturation phase, the biomass expands into confluent multilayers along with formation of the extracellular matrix (ECM) (12–30 h), and hyphae formation increases in the uppermost layers. The dispersion phase is characterized by release of yeast cells exclusively from the topmost region of biofilm to disperse to new sites [16]. The growth of *C. albicans* biofilms in in vivo catheter models subjected to transient (once or twice a day) low flow exhibited different structures than those grown under static conditions [7,17,18,19,20]. Particularly striking was that the foundational layer of biofilms formed under flow consisted of yeast, hyphae and pseudohyphae rather than the uniform layer of yeast cells at the base of statically formed biofilms [16]. In vivo biofilms of *Candida* formed in cases of oral candidiasis show an additional unique characteristic, namely the development of patches of infection [21]. 

Of the few in vitro biofilm studies performed with *C. albicans* under flow, only end-points of growth were measured, therefore understanding of the crucial events needed for biofilm growth and development is lacking [12,14,22]. In this study, we developed a novel system allowing real-time imaging of *C. albicans* biofilms under flow. This system permits quantitative measurement of the dynamics of biofilm development that up to now have not been assessed. We analyzed the rates of cell attachment and detachment to the substrate, rates of cell detachment independent of available biomass (normalized detachment rate), cell–cell adhesion relative to cell-surface adhesion, and the total biofilm growth rate to understand critical components and developmental stages of *Candida albicans* biofilms under flow. We found that biofilms formed in our flow system not only reproduced the mixed yeast, hyphae, and pseudohyphae layers of biofilms formed in catheter models, but also developed microcolonies from single *Candida albicans* hyphal cells that are very similar phenotypically to the microcolonies formed upon invasion of epithelial monolayers [23].

## 2. Materials and Methods

### 2.1. Strains

*Candida albicans* CAI4 *(URA+*) (Δ*ura3*::*imm434*/Δ*ura3*::*imm434 RPS1*/Δ*rps1*::*Clp10*-*URA3*) [24] was used as WT control. The hyperfilamentous *C. albicans* deletion mutant Δ*hog1* [25] was used to compare morphologies of biofilm formation. Cultures were grown overnight in 1% (*w*/*v*) yeast extract, 2 % (*w*/*v*) bacto peptone, and 2% (*w*/*v*) glucose (YPD; Difco, Detroit, MI, USA). Cell densities of overnight cultures were determined using a cytometer, and values were used to determine volumes of overnight culture to add to the attachment flask (described below) to reach 1 × 10^6^ cells/mL. After addition of culture, cells were allowed to acclimate for 15 m prior to initiation of flow.

### 2.2. Flow System

**Figure 1:**
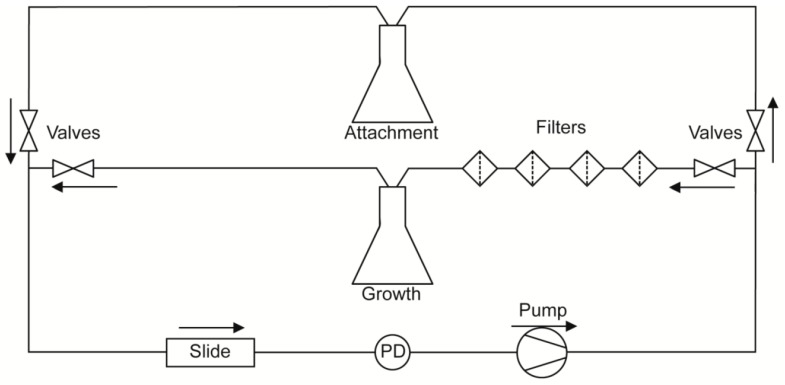
Schematic of Flow System. Cell-seeded media circulates from the attachment flask for the first 2 h, during which time both valves to and from the growth flask are closed. After 2 h, the valves to and from the attachment flask are closed and those of the growth flask are opened, allowing circulation of cell-free media for the remainder of the experiment (additional 16 h). Media is maintained as cell-free using four sequential filters. Arrows indicate direction of flow. PD: Pulsation Damper, used to reduce pulsation of slide surface caused by pump.

A diagram of the flow system used in this study is depicted in Figure 1. To separate *C. albicans* cell attachment from subsequent biofilm growth and development, and to allow evaluation of detachment independent of fresh cell seeding, flow experiments were split into two phases. During the first phase (attachment phase), fresh YPD seeded with *C. albicans* cells (1 × 10^6^ cells/mL) was circulated through a µ-Slide I 0.8 Luer family ibiTreat flow chamber (ibidi, Martinsried, Germany) using a Masterflex^®^ L/S^®^ variable speed pump (Cole-Parmer, Vernon Hills, IL, USA). This phase proceeded for 2 h, during which time cells were able to attach to the coverslip surface of the flow chamber. Afterwards, the source of media to the slide was switched to cell-free YPD for the remaining 16 h of the experiment (growth phase). The return flow during the growth phase was passed through four sequential cell filters: first two coarse filters (20 and 10 µm pore size; Analytical Scientific Instruments, Richmond, CA, USA), then a 2 µm pore size HPLC filter (Sigma Aldrich, St. Louis, MO, USA) followed by a 0.22 µm polyvinylidene fluoride filter (Sterivex™; Millipore, Billerica, MA, USA), before being recycled so as to prevent contamination of the stock medium. Thus, during the attachment phase, cells are allowed to re-circulate across the surface of the slide, but during the growth phase all cells are removed prior to re-circulation, and media to the slide remains cell-free for the rest of the experiment.

In all experiments, the flow was set to generate a shear force of 0.8 dynes/cm^2^ across the surface of the flow chamber. This value has been previously calculated as the approximate shear force that human saliva exerts on the tooth surface [9]. A hotplate stirrer with an external temperature probe was used to warm the media to 37 °C. For those experiments indicated as 37 °C the microscope, including the slide being imaged, and several feet of preceding tubing, were warmed to 37 °C, maintaining biofilm growth at this temperature, otherwise these were kept at room temperature (RT).

### 2.3. Imaging

All images were taken using a Zeiss AxioScope A.1 transmitted light microscope (Zeiss, Göttingen, Germany) using darkfield illumination, and acquired using the µManger software [26,27]. All imaging conditions (exposure time, light intensity, magnification, N.A., condenser height, and approximate positioning of the slide) were maintained between experiments. For all experiments, images were acquired every two minutes during the attachment phase, and every 15 min during the growth phase.

### 2.4. Image Analyses

All image analyses were performed in the ImageJ software environment [28] after conversion to an 8-bit grayscale file format. ImageJ macro scripts used for all analyses are functionally described below. Statistical analyses, including linear and non-linear regressions, were performed in Graphpad Prism^®^ version 5.03 software. 

To determine the coverage area of the biofilm, thresholds were applied to every image at a gray value minimum of 15, and percent surface area was measured. To evaluate the biomass of the attached cells (biofilm biomass), a densitometry analysis was performed. Specifically, the cumulative gray values of all pixels above 15 were evaluated for every frame of the darkfield time-lapse videos. The growth rate of each biofilm was then evaluated by linear regression of all biomass data collected. 

To evaluate the rate of cell attachment during the attachment phase, a given frame (n) was subtracted from its next frame (n + 1) for every image of the attachment phase ((n + 1) – n). This subtraction resulted in an image where any cell that attached to the imaging region between the frames remained bright (at their original intensity), while cells that remained constant between frames were removed from the image. A threshold was then applied to these calculated images, highlighting newly attached cells, and subsequently processed using the ImageJ binary erosion filter to limit background noise and minor shifts in cell position. The area of newly attached cells (µm^2^) was then determined on each of these images using the analyze particles tool in ImageJ. To increase specificity towards cells, particles had to be a minimum of 20 µm^2^. Rates of cell attachment were then determined by fitting the cumulative attachment area for the first 2 h with linear regressions.

Rate of cell detachment was determined in a similar manner to cell attachment, but the image subtraction was reversed (n – (n + 1)), resulting in an image that highlighted cells that detached between frames. Detachment rates were evaluated over the entire duration of the experiment (attachment and growth phases) in a manner similar to attachment rates. The rate of total cell detachment was found to be dependent on the biomass of the biofilm, but this simply arises due to the increased number of cells available to detach. Thus, values obtained for this variable did not reflect the relative ease with which cells are removed from the biofilm or substrate surface, which is the parameter we were interested in. Thus, we normalized the area of detachment obtained between each frame to the biomass of the biofilm prior to these detachments ((n – (n + 1))_area_/n_biomass_), resulting in a value that effectively represents the proportion of cells that detached (referred to as normalized detachment). The rates of normalized detachment were also evaluated using linear regressions, however data during the attachment phase was excluded from the analyses, as these values were often not stable, likely due to low biomass values. 

To estimate relative cell–cell to cell-surface binding strengths, we performed image subtraction ((n + 1) – n) to determine newly attached cells at each frame, and applied a threshold to these images (as described above). These images were then processed using the ImageJ binary erosion filter, and particles at least 20 µm^2^ and with a circularity value of at least 0.4 were counted as cells. These particles were then compared to images of the biofilm coverage area of the preceding frame (n), to determine regions of overlap (completed using the “AND” operator in the ImageJ image calculator). Regions of overlap were counted as cell–cell adhesion events if they were at least 2.5 µm^2^. The number of cell–cell adhesions was then normalized to the total number of adhesion events, giving the relative cell–cell adhesion. 

### 2.5. Full Slide Scans

All slide scans were conducted with a Bio-Rad GS-700 Imaging Densitometer (Bio-Rad, Hercules, CA, USA), and analyzed by densitometry analysis using ImageJ with no lower threshold applied.

### 2.6. Statistical Comparisons

For all rates determined through regression analyses, non-overlapping 95% confidence intervals were considered statistically significant at *p* < 0.05. For cell–cell adhesion, means ± S.D. of relative cell–cell adhesion between 1 and 2 h were determined and statistical significance was evaluated using a one-way ANOVA followed by a *post-hoc* Tukey’s *t*-test (significance at *p* < 0.05). 

## 3. Results

### 3.1. Biofilm Formation under Flow

**Figure 2:**
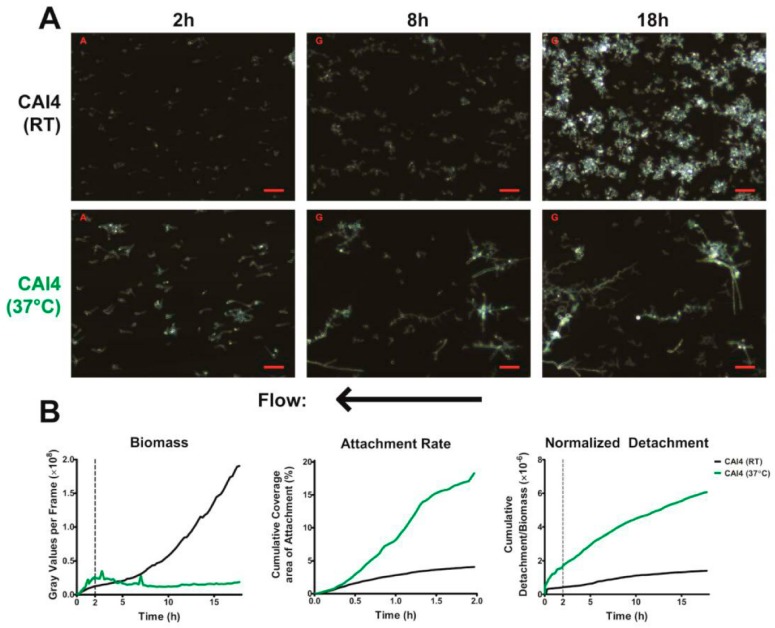
Biofilm Formation under Flow: (**A**) Representative darkfield images of biofilm formation under flow are shown for wild-type cells at room temperature (top, black) and 37 °C (middle, green) at 2, 8 and 18 h of growth. Scale bars indicate 50 µm. Arrow indicates direction of media flow for every image; (**B**) The total biomass within the imaging region (determined by densitometry analysis), the rate of cell attachment, and the detachment rate normalized to the biomass over time are shown. Data are means of *n* ≥3 experiments.

Our novel flow system is designed to separate and quantitate two phases of *C. albicans* biofilm development. The first phase is cell attachment that occurs while cells (1 × 10^6^ cells/mL) are circulated through the flow chamber with a shear force of 0.8 dynes/cm^2^ for 2 h to allow attachment to the substrate (Figure 2A—2 h). We chose to define the cell attachment phase to occur during the first 2 h to conform to literature values [4]; but also since 2 h provided a uniform, but low density, foundation of attached cells in both WT conditions (at RT and 37 °C). Following the attachment phase, a cell attachment rate was quantified by calculating the change in coverage area of attached cells as a percentage of total area over time; and cell–cell adhesion was quantified by calculating an average of the number of cell–cell adhesion events/total adhesion events. The second phase, biofilm growth and development, occurred over the next 16 h during which only media without cells was circulated through the chamber also with a shear force of 0.8 dynes/cm^2^ (Figure 2A—8 and 18 h). At the end of the growth phase, total biomass, biofilm growth rate (rate of biomass formation over time), and normalized cell detachment rate (proportion of detached cells compared to total biomass) were calculated. Values obtained for WT cells are shown in Table 1.

*C. albicans* WT cells readily attached to the substrate surface during the 2 h attachment phase under flow (Videos S1, S2). Interestingly, we observed that cells frequently rolled across the surface prior to attachment, similar to leukocytes rolling prior to endothelial attachment and diapedesis (Videos S5–S7). Germinated cells were found to frequently roll along their short axis, with their long axis perpendicular to the flow (Video S5). However, several differing angles of rolling were observed, including teetering while rolling, and rolling with the long axis parallel to the flow (i.e., flipping head over heels). Due to the difficulty of imaging adherence events at high speed, we were unable to determine the role that these different rolling characteristics may play in cell attachment. The attachment rate of *C. albicans* WT cells incubated at 37 °C was nearly 4-fold higher than *C. albicans* RT, however, this was largely countered by a ~4-fold increase in normalized detachment for the same period (Table 1 and Figure 2B). Cell–cell adhesion values were not different between RT and 37 °C, suggesting that temperature does not have a large impact on this process. Adherent yeast cells maintained at RT then began to proliferate to form small colonies of cells that were primarily clusters of blastospores; therefore, by 8 h, biofilm was comprised of uniformly distributed small colonies (5–15 cells). By 18 h, these colonies expanded to roughly 20–500 cells with diameters generally between 30 and 200 µm. This range was due to colonies merging together and new colonies continuing to form from cells that had detached upstream of the imaged region. However, hyphal cells maintained at 37 °C exhibited very different biofilm morphology than yeast cells at RT. Hyphal projections (often multiple hyphae) grew very robustly from single attached cells along the substrate surface and these hyphae were profusely budded (Figure 2A—8 h). During the early growth phase of WT cells at 37 °C, surface detachment of hyphal cells as well as detachment of yeast cells budding off hyphae both occurred; therefore, by 5 h, the total biomass of 37 °C hyphal biofilm was equal to RT biofilm (Figure 2B—left). Analyses of normalized detachment during early biofilm growth showed that the normalized detachment of WT cells at 37 °C was approximately 5-fold greater at 5 h (Figure 2B—right) as compared to cells at RT. For reasons discussed below, we found that analyses of biofilm formation at 37 °C beyond this point were likely not truly illustrative of biofilm formation using this methodology. 

**Figure 3:**
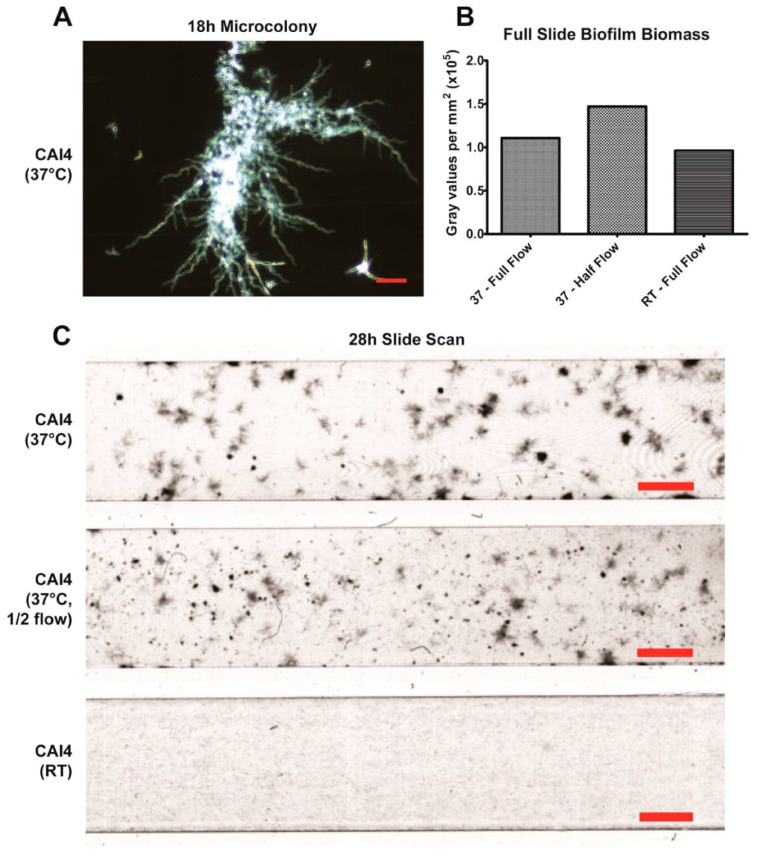
Formation of Microcolonies under Flow at 37 °C: (**A**) Representative image of a microcolony from a single hyphae, showing extensive hyphal branching, is shown (top left, scale bar indicates 50 µm); (**B**) Biomass comparisons between slides at 37 °C (full flow at 0.8 dynes/cm^2^, and half flow at 0.4 dynes/cm^2^) and room temperature were done through densitometry analysis at 28 h of growth using a flatbed scanner (the heterogeneity of the 37 °C slides prevents traditional microscope analysis); (**C**) Images of scanned biofilms after 28 h of growth are shown. Scale bars indicate 2 mm.

Unexpectedly, we observed that hyphal biofilms formed at 37 °C developed distinct microcolonies (Figure 3A; frequently >400 µm diameter) that were interspersed with large regions containing only smaller clusters or individual cells. We discovered through direct observation that these microcolonies can develop from only a single branching hyphae cell (Video S3). However, this very heterogeneous microcolony formation by 37 °C WT cells prevented quantitation of biofilm formation across the entire slide when imaging such finite and random regions using our methodology. Thus, we found that comparative experiments between multiple *C. albicans* strains were best carried out at RT, as this generates a more homogenous biofilm.

In all of our quantifiable microscopy experiments performed, we only obtained data from the sparse regions in between microcolonies, skewing the results illustrated in Figure 2. Therefore, in an effort to make a more accurate comparison of biofilm formation between RT and 37 °C slides, and to better visualize the heterogeneous nature of slides at 37 °C, we performed end-point scans of the whole slides following 28 h of continuous flow (Figure 3B,C; 2 h attachment phase then 26 h growth phase). At this point, the microcolonies of the 37 °C slides were large enough to be easily visualized with the unaided eye. These scans clearly illustrate the large gaps between microcolonies that can lead to non-representative results. We also attempted to lower the flow rate (0.4 dynes/cm^2^) at 37 °C in an effort to generate a more homogenous biofilm. While this did result in more microcolonies with less spacing, the biofilm was still not homogenous enough to generate representative data using our microscopy system.

### 3.2. Effects of Hyperfilamentation on Biofilm Formation

**Figure 4:**
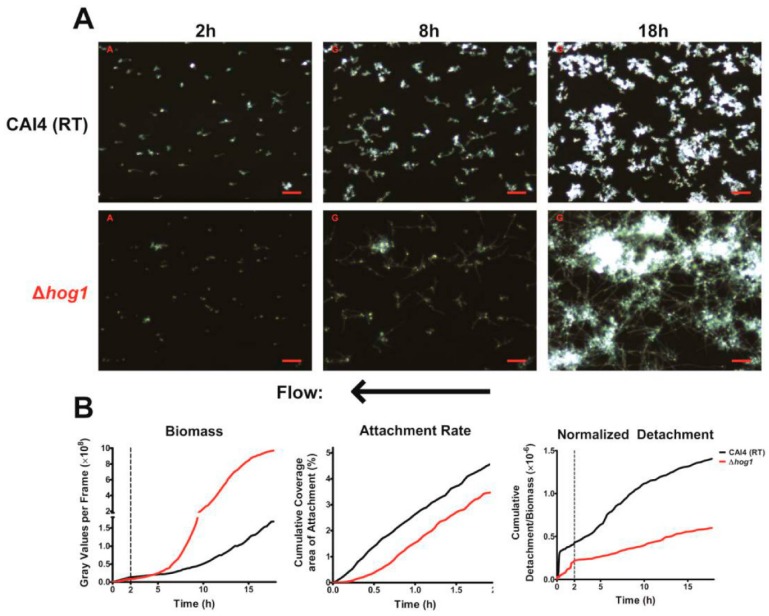
Biofilm Formation of the Hyperfilamentous mutant Δ*hog1* under flow: (**A**) Representative darkfield images of biofilm formation under flow are shown for wild-type (**top**) and Δ*hog1* (**bottom**) cells grown at room temperature at 2, 8 and 18 h of growth. Scale bars indicate 50 µm. Arrow indicates direction of media flow for every image; (**B**) The total biomass within the imaging region (determined by densitometry analysis), the rate of cell attachment, and the detachment rate normalized to the biomass over time are shown for Δ*hog1* biofilms (red) and wild-type at room temperature biofilms (black). Data are means of *n* ≥3 experiments.

To differentiate between the roles of filamentation and temperature for biofilm formation, we assessed the ability of a *C. albicans* hyperfilamentous mutant, Δ*hog1*, to form biofilms at RT (Figure 4, Video S4). *C. albicans* Δ*hog1* mutants (that have a hyperfilamentous phenotype due to de-repression of Brg1 [29] unrelated to temperature) showed sustained hyphal elongation at RT. These cells were predominantly yeast-form during attachment to the slide surface and initiated germ tube formation shortly after the start of the experiment (showing that substrate binding may also be a signal for hyphal initiation in Δ*hog1* cells). 

*C. albicans* Δ*hog1* cells had slightly (but significantly) reduced substrate attachment rates compared to WT cells, and no difference in their cell–cell adhesion (Figure 4B—center, Table 1). Although the total biomass of Δ*hog1* biofilms was equivalent to WT at early times (Figure 4B—left, until 5 h), the biofilm biomass was significantly greater than WT biofilm after 10 h growth. The Δ*hog1* biofilm was characterized by extensive cell germination and branching morphology, similar to cells at 37 °C, but attached cells were more prevalent and homogeneous, resulting in a very dense mesh-like network of hyphae. These results showed that filamentation per se is not a requirement for microcolony formation, rather there are specific transcriptional programs activated by temperature that influence microcolony formation. Furthermore, the endpoint biofilm biomass of *C. albicans* Δ*hog1* cells was at least 5-fold more and the biofilm growth rate at least 7-fold more than that of WT cells; while the normalized detachment rate was reduced by half (Figure 4B, Table 1). These quantitative results show that the reduced normalized detachment rate was the primary factor responsible for the increased biomass of Δ*hog1* biofilm, since the substrate attachment rate was lower than WT cells, and cell to cell adhesion values were no different than WT cells.

## 4. Discussion

In this study, we were able to develop a novel system to analyze in real-time the attachment and development of *C. albicans* biofilms under flow. With this system, we were able to analyze the first 18 h of biofilm development under several different conditions for unique quantitative measures of biofilm development, including the cell attachment rate, detachment rate, relative cell–cell adhesion and biofilm biomass over time. To the best of our knowledge, this is the first time that cell attachment and detachment of biofilm forming microbes has been measured in this manner.

Our results support a previous study that showed that the dispersion of *C. albicans* is a continuous process (Figure 2B—right) [14]. Cells detached from our biofilm throughout their growth and development, with the number of detaching cells increasing with increasing biofilm biomass. This is in contrast to the process of dispersion in bacterial cells, where they exhibit discontinuous large-scale dispersals by forming pillar and mushroom structures that ultimately release numerous bacteria upon maturation. As the process of dispersion was constant, and was found to be dependent on the available biomass, we normalized the detachment to the total biofilm biomass at each frame, permitting measurement of how easily cells were being removed from the biofilm (normalized detachment rate). We also found that the normalized detachment rate remains relatively constant for the first 18 h of biofilm development under flow. This was a surprising finding, since dispersion is considered a property of mature biofilms. However, our results show that cells under constant laminar flow undergo continuous detachment and seeding, and that these conditions may be more representative of the development of in vivo biofilms. 

Using our imaging system at 37 °C, we were successfully able to observe the development of two major phenotypic characteristics of biofilms grown under flow. The first being that these biofilms formed mixed yeast, hyphae, and pseudohyphae basal layers (Figure 2A), which has been previously observed in flow catheter models of biofilm formation [7,17,18,19,20]. This mixture seems to largely be the result of hyphae growing along the substrate surface, branching and forming laterally budded yeast and pseudohyphae. Additionally, flow-generated biofilms formed distinct microcolonies (Figure 3A,C), similar to those found within tongue plaques in in vivo infection [30]. These microcolonies evolved from single hyphal cells that branched extensively, forming vast tree-like structures.

In addition to these two major characteristics, we also found that most hyphae (from both WT cells grown at 37 °C or Δ*hog1* cells grown at RT) grew in a sinusoidal morphology under flow conditions. This morphology has been observed previously, most notably when *C. albicans* cells were grown on cellophane overlying a 2% agar base [31,32]. Previous studies identified these structures as 3-dimensional helices; however, upon closer examination, we found that the hyphae in our experiments were predominantly lying flat along the surface while growing in a sinusoidal fashion. It is likely that this morphology may be a result of laminar flow, but it is unclear if this hyphal morphology is also found in vivo. Interestingly, helical hyphae were found to be more resistant to antifungals than their straight hyphae counterparts [31].

Flow rates can affect biofilm formation by both altering the shear force exerted on the biofilm and by altering the rate at which fresh nutrients are delivered to the cells. The latter is determined by the bulk flow velocity (cm/min) of the fluid, which can be calculated by dividing the flow rate (mL/min) by the cross-sectional surface area of the channel (cm^2^). Alterations to the flow rate will also alter both the shear force and the flow velocity, and can be used to model changes in salivary flow or to model other settings of fungal biofilm development such as catheters. It is possible to isolate the effects of variations in shear force or flow velocity independently from one another by adjusting both the flow rate and the cross-sectional surface area simultaneously. The slides used in our study are available in multiple channel heights, thus one parameter can be set as a constant (e.g., shear force = 0.8 dynes/cm^2^) across multiple channel heights by adjusting the flow rate. Thus, factors limiting biofilm development at a particular flow rate (either acquiring adequate nutrients in low flow velocity vs. maintaining adhesion in high shear force) can be determined.

Nutrient availability in our study is likely slightly affected by our use of a recirculating media system, which would also retain and recirculate cell signaling molecules. However, given that we saw continued cell division, and hyphal growth at >30 h (data not shown), we do not believe this is a major factor in our experiments. For prolonged, multi-day experiments, it would be possible to modify the system to continuously supply fresh nutrients, as described previously [33].

The use of darkfield microscopy with our system is advantageous over the use of fluorescently labeled cells since there is no photo-bleaching. Additionally, fluorescence microscopy typically requires longer exposure times and higher intensity lighting that can cause phototoxicity [34]. While fluorescent microscopy does have many tools available to assist in image analysis, particularly COMSTAT [35], we were able to develop our own algorithms that took advantage of several features of darkfield microscopy. Traditional microscopy (no optical sectioning), as was used here, is power conservative, meaning that objects which are out of focus contribute nearly the same number of photons to an image as they would if they were in focus [36]. In this system, cells that are further from the surface are still contributing to the analyses, therefore the contribution of all cells in the 3D biofilm are calculated despite capturing only a single 2D image at each time point.

Furthermore, this flow system can be easily adapted to study the effects of antimicrobial agents on biofilms by placement of an upstream in-line injection port for drug delivery. Also, epithelial or endothelial cells can be grown inside the slide channel prior to introduction of microbial cells to study fungal cell attachment, growth, and cell invasion in real time. This flow system can be assembled without use of custom manufactured parts and is reasonably inexpensive. The use of darkfield microscopy also allows images to be acquired with relatively simple microscopes, and allows seamless analysis of multiple microbial organisms. The versatility and adaptability of our flow system enable it to be used to study many different potential phenomena related to biofilm development.

## Figures and Tables

**Table 1:** Quantification of real-time biofilm formation of *Candida albicans* under dynamic flow.

Strain (Temperature)	Attachment Rate ^1^	Cell–Cell Adhesion ^2^	Biomass ^3^ (×10^6^)	Biofilm Growth Rate ^4^ (×10^6^)	Normalized Detachment Rate ^5^ (×10^−8^)
CAI4 (RT)	2.43	0.46	190.5	11.43	4.29
CAI4 (37 °C)	9.27	0.62^NS^	ND	ND	ND
Δ*hog1* (RT)	1.71	0.41^NS^	969.4	73.75	2.67

Each value represents a mean or best-fit slope of *n* ≥3 experiments. NS indicates no significant difference between each strain compared to CAI4 at room temperature (RT). All other values were significant (*p* < 0.05). ND indicates that the value was not determined. ^1^ Attachment Rate represents the average coverage area of newly attached cells/imaging area/h. Values represent a change in coverage area of attached cells as a percentage of total area over time. ^2^ Cell–Cell Adhesion was calculated as the average events/total substrate adhesion events between 1 and 2 h during the attachment phase. ^3^ Biomass was calculated as the cumulative light intensity of the imaging area (in gray values), measured at 18 h. ^4^ Biofilm Growth Rate was calculated as the best fit slope of the biomass over time in gray values/h obtained by linear regression. ^5^ Normalized Detachment Rate was calculated as the average total detachment rate (average area of newly detached cells/total imaging area/h)/biomass. Values indicate the probability for any individual cell to detach from the surface or biofilm.

## Supplementary Materials

The following are available online at www.mdpi.com/2309-608X/3/1/13/s1, Video S1: Biofilm formation of WT cells at RT, Video S2: Biofilm formation of WT cells at 37 °C, Video S3: Microcolony formation from a single hyphal cell, Video S4: Biofilm formation of Δ*hog1* cells at RT, Video S5: Cell rolling perpendicular to the flow, Video S6: Cell attaching to the substrate, Video S7: Cell-cell attachment event.
