# Supplementary material for: Real-Time Approach to Flow Cell Imaging of Candida albicans Biofilm Development
Source: J Fungi (Basel). 2017 Mar 6;3(1):13. doi: 10.3390/jof3010013 (PMC5715965; doi:10.3390/jof3010013)
Supplement: Supplementary file 1 [file jof-03-00013-s001.zip › jof-176102-suppl/4.jof-176102-suppl.-legend.pdf]

Supplementary Materials: Real-Time Approach to Flow Cell Imaging of *Candida albicans* Biofilm Development

Andrew McCall and Mira Edgerton

Supplemental Movie 1: Biofilm formation of WT cells at RT. This time-lapse darkfield microscopy video shows the attachment of WT cells to the substrate during the attachment phase (indicated by a "A" in the upper left; images acquired every 2 m), followed by the subsequent growth and development of the biofilm during the growth phase (indicated by a "G" in the upper left; images acquired every 15 m). Cell-seeded media (1×10^6) was used during the attachment phase, while cell-free media was used during the growth phase. Flow is from the right to left. Scale bar indicates 50 µm.

Supplemental Movie 2: Biofilm formation of WT cells at 37 °C. This time-lapse darkfield microscopy video shows the attachment of WT cells to the substrate during the attachment phase (indicated by a "A" in the upper left; images acquired every 2 m), followed by the subsequent growth and development of the biofilm during the growth phase (indicated by a "G" in the upper left; images acquired every 15 m). Cell-seeded media (1×10^6) was used during the attachment phase, while cell-free media was used during the growth phase. Flow is from the right to left. Scale bar indicates 50 µm.

Supplemental Movie 3: Microcolony formation from a single hyphal cell. Time-lapse darkfield microscopy video showing the growth and development of a microcolony from a single mother cell (center of frame), beginning from the 5 h mark (images acquired every 15 m). Flow is from the right to left. Scale bar indicates 50 µm.

Supplemental Movie 4. Biofilm formation of Δ*hog1* cells at RT. This time-lapse darkfield microscopy video shows the attachment of Δ*hog1* cells to the substrate during the attachment phase (indicated by a "A" in the upper left; images acquired every 2 m), followed by the subsequent growth and development of the biofilm during the growth phase (indicated by a "G" in the upper left; images acquired every 15 m). Cell-seeded media (1×10^6) was used during the attachment phase, while cell-free media was used during the growth phase. Flow is from the right to left. Scale bar indicates 50 µm.

Supplemental Movie 5: Cell rolling perpendicular to the flow. This time-lapse darkfield microscopy video shows the rolling behavior of a germ tube perpendicular to the direction of flow. Flow is from the right to left. Time interval from start is indicated in lower left. Scale bar indicates 10 µm.

Supplemental Movie 6. Cell attaching to the substrate. This time-lapse darkfield microscopy video shows the rolling behavior of a cell cluster prior to its attachment to the substrate surface. Flow is from the right to left. Time interval from start is indicated in lower left. Scale bar indicates 10 µm.

Supplemental Movie 7: Cell–cell attachment event. This time-lapse darkfield microscopy video shows the rolling behavior of a cell cluster prior to its attachment to another already adhered cell. The force of this attachment causes the already adhered cells to rotate slightly. Flow is from the right to left. Time interval from start is indicated in lower left. Scale bar indicates 10 µm.
